# Data-driven retrieval of population-level EEG features and their role in neurodegenerative diseases

**DOI:** 10.1093/braincomms/fcae227

**Published:** 2024-07-31

**Authors:** Wentao Li, Yogatheesan Varatharajah, Ellen Dicks, Leland Barnard, Benjamin H Brinkmann, Daniel Crepeau, Gregory Worrell, Winnie Fan, Walter Kremers, Bradley Boeve, Hugo Botha, Venkatsampath Gogineni, David T Jones

**Affiliations:** Department of Neurology, Mayo Clinic, Rochester, MN 55905, USA; Department of Neurology, Kaiser Permanente Northern California, Sacramento, CA 95758, USA; Department of Neurology, Mayo Clinic, Rochester, MN 55905, USA; Department of Bioengineering, University of Illinois, Urbana, IL 61801, USA; Department of Computer Science & Engineering, University of Minnesota, Minneapolis, MN 55455, USA; Department of Neurology, Mayo Clinic, Rochester, MN 55905, USA; Department of Neurology, Mayo Clinic, Rochester, MN 55905, USA; Department of Neurology, Mayo Clinic, Rochester, MN 55905, USA; Department of Neurology, Mayo Clinic, Rochester, MN 55905, USA; Department of Neurology, Mayo Clinic, Rochester, MN 55905, USA; Department of Quantitative Health Sciences, Mayo Clinic, Rochester, MN 55905, USA; Department of Quantitative Health Sciences, Mayo Clinic, Rochester, MN 55905, USA; Department of Neurology, Mayo Clinic, Rochester, MN 55905, USA; Department of Neurology, Mayo Clinic, Rochester, MN 55905, USA; Department of Neurology, Mayo Clinic, Rochester, MN 55905, USA; Department of Neurology, Mayo Clinic, Rochester, MN 55905, USA

**Keywords:** EEG, tensor decomposition, neurodegenerative disease, PET, fluid biomarkers

## Abstract

Electrophysiologic disturbances due to neurodegenerative disorders such as Alzheimer’s disease and Lewy Body disease are detectable by scalp EEG and can serve as a functional measure of disease severity. Traditional quantitative methods of EEG analysis often require an a-priori selection of clinically meaningful EEG features and are susceptible to bias, limiting the clinical utility of routine EEGs in the diagnosis and management of neurodegenerative disorders. We present a data-driven tensor decomposition approach to extract the top 6 spectral and spatial features representing commonly known sources of EEG activity during eyes-closed wakefulness. As part of their neurologic evaluation at Mayo Clinic, 11 001 patients underwent 12 176 routine, standard 10–20 scalp EEG studies. From these raw EEGs, we developed an algorithm based on posterior alpha activity and eye movement to automatically select awake-eyes-closed epochs and estimated average spectral power density (SPD) between 1 and 45 Hz for each channel. We then created a three-dimensional (3D) tensor (record × channel × frequency) and applied a canonical polyadic decomposition to extract the top six factors. We further identified an independent cohort of patients meeting consensus criteria for mild cognitive impairment (30) or dementia (39) due to Alzheimer’s disease and dementia with Lewy Bodies (31) and similarly aged cognitively normal controls (36). We evaluated the ability of the six factors in differentiating these subgroups using a Naïve Bayes classification approach and assessed for linear associations between factor loadings and Kokmen short test of mental status scores, fluorodeoxyglucose (FDG) PET uptake ratios and CSF Alzheimer’s Disease biomarker measures. Factors represented biologically meaningful brain activities including posterior alpha rhythm, anterior delta/theta rhythms and centroparietal beta, which correlated with patient age and EEG dysrhythmia grade. These factors were also able to distinguish patients from controls with a moderate to high degree of accuracy (Area Under the Curve (AUC) 0.59–0.91) and Alzheimer’s disease dementia from dementia with Lewy Bodies (AUC 0.61). Furthermore, relevant EEG features correlated with cognitive test performance, PET metabolism and CSF AB42 measures in the Alzheimer’s subgroup. This study demonstrates that data-driven approaches can extract biologically meaningful features from population-level clinical EEGs without artefact rejection or a-priori selection of channels or frequency bands. With continued development, such data-driven methods may improve the clinical utility of EEG in memory care by assisting in early identification of mild cognitive impairment and differentiating between different neurodegenerative causes of cognitive impairment.

## Teaser

Li and Varatharajah *et al*. report that data-driven tensor decomposition approach to EEG analysis can automatically extract biologically meaningful brain features including posterior alpha and anterior delta/theta rhythms, which correlate with cognitive test performance, PET metabolism and CSF Amyloid-Beta 42 measures, and can distinguish Alzheimer’s and Lewy Body dementia patients from controls.

## Graphical Abstract

**Graphical abstract:**
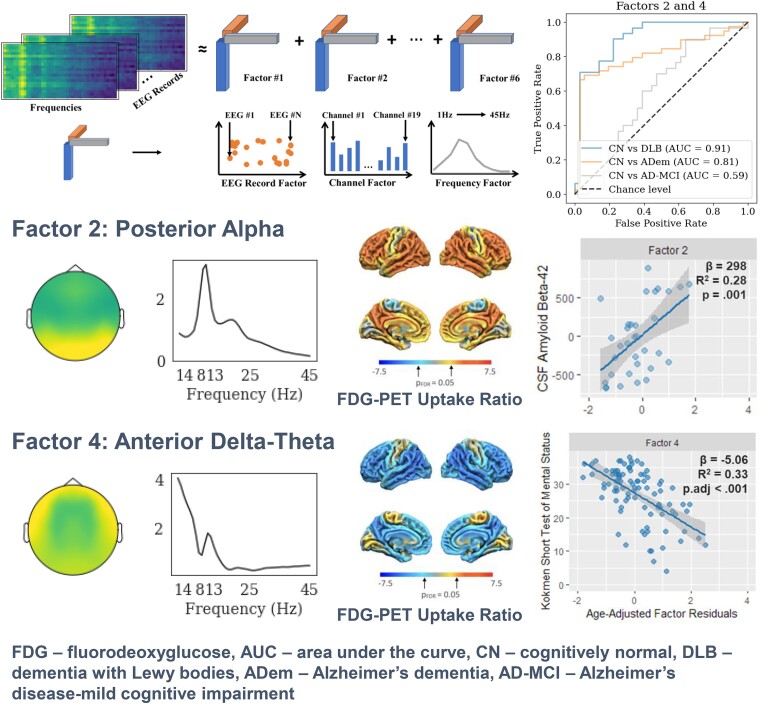

## Introduction

In neurodegenerative disorders such as Alzheimer’s disease (AD) and dementia with Lewy Bodies (DLB), the pathophysiology involves an interaction between cellular pathways and large-scale network physiology related to coordinated function in ensembles of neurons that support mental functioning.^1-4^ This large-scale disturbance in activity in neuronal ensembles is accompanied by changes in electrophysiology detectable by scalp EEG before changes on structural brain imaging such as CT or MRI are detectable.^5,6^ This is strikingly apparent in DLB, in that this condition is not characterized by significant changes in measures of brain structure but has marked disturbance in measures of electrophysiology. This contrast is reflected in the most recent diagnostic criteria for DLB.^7^ Neurodegeneration-associated EEG changes during the mild cognitive impairment (MCI) and dementia stages have also been shown to correspond with cognitive performance and disease severity based on functional imaging and fluid biomarkers.^8,9^

Compared to other clinically available tests for neurodegenerative cognitive impairment, such as CSF analysis, neuropsychologic evaluations, or fluorodeoxyglucose (FDG) and molecular PET, scalp EEGs are noninvasive and relatively inexpensive tests available at most neurology practices worldwide.

Quantitative analysis of EEG has been extensively explored in the context of characterizing and diagnosing neurodegenerative diseases. This includes differences between AD Dementia (ADem), AD-MCI and healthy controls using resting-state EEG-derived features.^10,11^ Furthermore, EEG has been shown to be useful in the differential diagnosis between AD, cerebrovascular diseases,^12,13^ DLB^7,9,14,15^ and Frontotemporal Lobar Degeneration spectrum of disorders.^16^ In all these studies, EEG signal power analysis has been the most common approach using the five major frequency bands, namely, delta (δ) 0.1–4 Hz, theta (θ) 4–8 Hz, alpha (α) 8–12 Hz, beta (β) 12–30 Hz and gamma (γ) > 30 Hz. Some studies have analyzed EEG synchrony measures within the above frequency bands and shown that disruptions in EEG-based measures of synchrony can differentiate between subjects with neurodegenerative symptoms and healthy individuals.^17^ Other EEG features including entropy,^18^ fractal dimension^19^ and the Lyapunov exponent^20^ have also been explored, but are not widely used.

Brain rhythms categorized by the five major frequency bands convey different information about brain function and connectivity.^21^ However, analyses using those bands provide only isolated views of brain activity within the corresponding frequency ranges and limit the ability to identify EEG patterns that may span several spectral bands. Furthermore, clinical EEG data may contain artefacts, which may confound EEG power or synchrony measures.

In this study, we investigate a data-driven approach based on tensor decomposition to automatically discover different constituents that contribute to EEG measured on the scalp without limiting the discovery to predefined frequency ranges or EEG channels.^22,23^ We applied this approach on the raw power spectral densities of a large sample of 12 176 routine scalp EEG recordings obtained in routine clinical setting within a broad frequency range (1 Hz—45 Hz) across all EEG channels (19 channels according to the 10–20 system). This approach allowed us to quantify multiple EEG features related to normal aging, neurodegenerative pathology and artefacts. Using the relevant features, we then characterized a subset of patients meeting clinical consensus criteria for AD-MCI (*N* = 30),^24^ ADem (*N* = 39),^25^ or DLB(*N* = 31),^7^ along with 36 age-matched cognitively normal subjects. Finally, we correlated the automatically extracted EEG features with their clinical diagnoses, cognitive test scores, CSF AD biomarker measures and fluorodeoxyglucose-positron emission tomography (FDG-PET) standardized uptake value ratios (SUVR).

Our model demonstrates that data-driven tensor decomposition of routine clinical EEGs can extract biologically meaningful effects without a-priori selection of frequency bands or channel sites. Moreover, these factors show good differentiation between patients with cognitive impairment due to AD and LBD and normal controls at the group level. Future iterations will aim at improving the diagnostic accuracy at the single subject level, particularly in the MCI and mild dementia stages of these diseases. Standardized quantitative analysis of EEG features has great potential to enhance the clinical utility of routine EEGs in cognitive neurology and to provide a widely available and inexpensive tool that can assist in the diagnosis and management of diseases such as AD and LBD.

## Materials and methods

### Dataset

We analyzed 12 176 clinical EEG recordings of 11 001 adult patients (≥18 years old) who underwent routine clinical EEG study at the Mayo Clinic, Rochester, between the years 2011 and 2021. The EEGs were recorded using the XLTEK EMU40EX headbox (Natus Medical Inc., Oakville, Ontario, Canada) according to the standard 10–20 localization system^26^ at a sampling rate of 256 Hz. This dataset is representative of the patient population that is referred for routine EEG at Mayo Clinic in Rochester, MN, which includes a wide range of neurologic and non-neurologic conditions including epilepsy, cognitive impairment, episodic migraines, cardiogenic syncope and functional spells.

### EEG visual review

The EEG records were visually reviewed by board-certified epileptologists and graded based on the Mayo Clinic EEG classification system: normal (no visible abnormalities), Dysrhythmia 1 (mild slowing), Dysrhythmia 2 (moderate to severe slowing) or Dysrhythmia 3 (epileptiform abnormalities).^27^ These grades along with full EEG reports were retrieved from the clinical EEG reporting database. We then assigned the above grades to binary labels as follows: (i) we combined normal and Dysrhythmia 1 grades to form ‘normal’ label; and (ii) we combined Dysrhythmia 2 and Dysrhythmia 3 grades to form ‘abnormal’ label.

### EEG preprocessing

EEG records were first converted to the Multiscale Electrophysiology Format (MEF).^28^ Using the Python programming language, the raw EEG timeseries, channel names and EEG sampling rate were extracted from the MEF records and subsequently processed using the MNE library.^29^ The EEGs were bandpass-filtered within 0.5–45 Hz and multiple 10s-long eyes closed awake (EC) epochs (between 4 and 6 epochs per participant) were selected using an automated algorithm. The automated algorithm consisted of the following steps: (i) divide the preprocessed EEG recording into epochs of 10 seconds; (ii) automatically score sleep stages using a previously published algorithm^30^; (iii) select epochs that do not contain eye blinks from the epochs scored as ‘awake’ by the sleep staging algorithm^29^; (iv) rank those eyes-closed epochs in order of spectral power in the alpha frequency range (8–12 Hz) in posterior channels (O1 and O2); and (v) select the top six epochs (fewer if six epochs were not available) with the highest posterior alpha power.^31^ In subsequent analyses, we kept only the 19 standard EEG channels according to the 10–20 system (i.e. Fp1, F3, F7, C3, T7, P3, P7, O1, Fp2, F4, F8, C4, T8, P4, P8, O2, Fpz, Fz, Cz and Pz) and excluded the sub-temporal EEG channels (Tp11 and Tp12) and channels corresponding to EKG activity, SpO2 and stimulation, as they were not recorded in all EEG studies.

### Power spectral measures

For each epoch, we then estimated the power spectral density (PSD, in decibels) at frequencies between 1 and 45 Hz (45 integer frequencies) using the Welch fast Fourier transform approach^32^ for all 19 EEG channels. We then averaged the PSD measures of each EEG record among all the identified epochs to obtain a single PSD vector for each channel. The PSD measures of each EEG record can now be represented as a matrix with shape 19 × 45 (19 channels and 45 frequencies).

### Tensor formation

The PSD matrices of all EEG records were stacked to form a 3D tensor (EEG records, channels, frequencies). Then all values in this tensor were normed to be within the 0–1 range by subtracting the minimum value and then dividing by the range of values. The dimensions of our final tensor were 12 176 × 19 × 45.

### Canonical polyadic (CP) decomposition

The canonical polyadic decomposition or the PARAFAC decomposition^33^ approximates a tensor with a sum of *R* rank-one tensors, where *R* is the rank and the resulting number of factors. Mathematically, a CP decomposition of a 3D tensor *T* with rank *R* can be written as follows:


$$T\approx \overset{R}{\underset{r=1}{\sum }}\;A_{r}⊗B_{r}⊗C_{r}$$

Here ⊗ denotes an outer product and *A_r_*, *B_r_* and *C_r_* are rank-1 tensors with shapes matching each of the three dimensions of *T*. The combination of *A_r_*, *B_r_* and *C_r_* is called a factor. The optimal *A_r_*, *B_r_* and *C_r_* are found using an optimization procedure known as the alternating least squares approach.^34^ Variations of the standard CP decomposition including nonnegativity constraints can be utilized with nonnegative input data.^35^ Our analysis utilized the tensortools Python library for performing tensor decomposition.^36^

### Tensor decomposition

**Figure 1:**
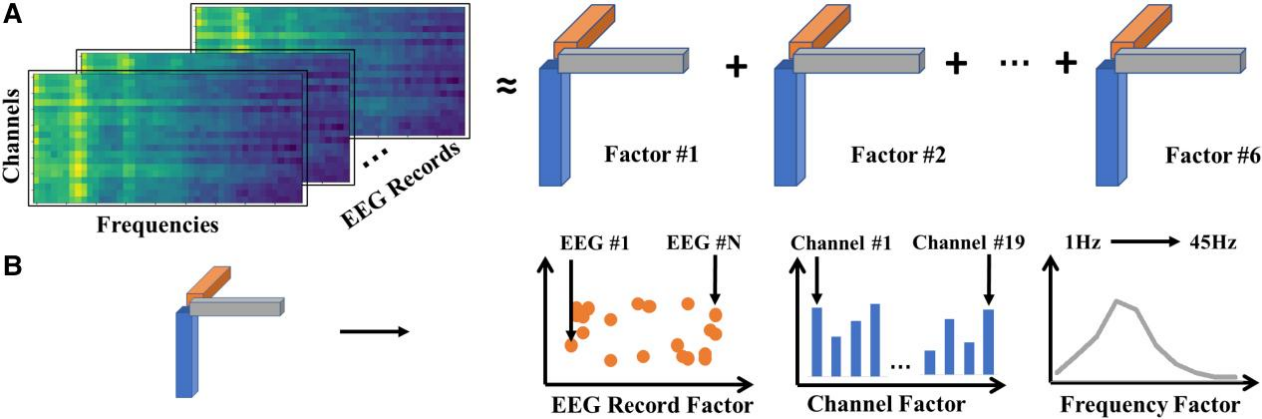
**Tensor decomposition of EEG power spectral densities.** We formed a 3D tensor by stacking the PSD matrices of all the EEG records (channels × frequencies × EEG records). (**A**) We then performed a nonnegative canonical polyadic decomposition on the above-formed tensor to identify six factors that approximated the original tensor. (**B**) Each factor consists of three rank-1 tensors corresponding to the channel, frequency and EEG record dimensions.

We performed a nonnegative canonical polyadic decomposition on the above-formed tensor to yield six factors representing electrophysiological features that best approximated the original tensor (Fig. 1A). We determined six to be a rational choice for the number of factors based on visual inspection of the identified factors, which demonstrated meaningful independent electrophysiological features. Fewer factors tended to combine some of these independent features, whereas additional factors tended to divide them into less meaningful features. Each factor consisted of 3 rank-1 tensors corresponding to the EEG record, channel and frequency dimensions (Fig. 1B). While the rank-1 tensors corresponding to the channel and frequency dimensions characterize the spatial and frequency patterns of brain activity, the rank-1 tensor corresponding to the EEG record dimension allows us to characterize group differences between patients/EEG records. Hence, we treat the tensor of the EEG record dimension as the weights/factor loadings of individual EEG records on the brain activity patterns characterized by the tensors corresponding to the channel and frequency dimensions. In subsequent analyses, we use the factor loading values to represent each EEG record in a lower dimensional space (i.e. loadings corresponding to six factors).

### Headplots

We visualized the rank-1 tensor corresponding to the channel dimension using the topographical headplots for visual interpretation of the spatial distribution of brain activity characterized by each factor.

### Controlling for age

Our subsequent analyses utilized the weights (rank-1 tensor corresponding to the EEG record dimension) for each factor to characterize group differences. Using linear regression, for each factor, we regressed the weights on age at the time of EEG and took the residuals as updated weights to control for the effect of age.

### Identification of patients with cognitive pathology and controls

A text string search of the EEG reports for ‘cognitive impairment’, ‘MCI’, ‘dementia’, ‘Alzheimer’, ‘AD’, ‘DLB’, ‘LBD’ and ‘Lewy’ was used to identify possible patients with cognitive impairment due to AD or LBD pathology. These cases were reviewed by a behavioural neurologist blinded to the EEG reports and extracted features, and who independently verified the patient’s diagnosis of either AD-MCI (*n* = 30), ADem (*n* = 39) or DLB (*n* = 31) based on documented history and clinical exam and established consensus criteria (i.e. the 2011 National Institute on Aging-Alzheimer's Association (NIA-AA) diagnostic guidelines for Alzheimer's disease^25^ and MCI,^24^ and the 2017 Fourth consensus report of the DLB Consortium^7^).

A score of <30 on the Kokmen Short Test of Mental Status (STMS), which is a measure of cognitive performance used routinely in the clinical evaluation of cognitively impaired patients,^37^ was used as the cutoff between ADem and AD-MCI.^38^ Because these diagnoses based on consensus criteria were made based on clinical history and exam and lacked confirmatory biomarker support (e.g. CSF biomarkers, amyloid PET, dopamine transporter imaging), subgroup analyses were considered exploratory for the purposes of this study. Moreover, using the consensus criteria creates an artificial dichotomy of clinical AD-MCI/Dementia versus DLB, whereas in reality comorbid AD and LBD pathology occurs in 30–75% of pathologically confirmed cases at autopsy.^39^ In cases of clinical ADem and AD-MCI patients who received CSF AD biomarker testing, an abnormal amyloid beta-42 (AB42) (≤ 1026 pg/ml) and/or abnormal phosphorylated-tau (pTau)/AB42 ratio (>0.023) were considered supportive of underlying Alzheimer’s disease pathology (*n* = 23).^40^

For the control group, 342 additional subjects between the ages of 50 and 90 were randomly selected from the overall EEG cohort and their charts were reviewed by a behavioural neurologist who was blinded to their EEG reports and features. As these were patients who had clinical reasons for obtaining an EEG and as most did not have recorded cognitive exam scores, a stringent list of exclusion criteria for potential CNS abnormalities was used as a surrogate for approximating a cognitively normal (CN), age-matched cohort.

Exclusion criteria included objective or subjective cognitive concerns (even with normal imaging and mental status exam scores) or functional neurologic disorders, presence of brain lesions such as stroke, malignancy (or brain/whole body radiation), or traumatic brain injury, diagnosis or strong clinical suspicion of epilepsy (regardless of EEG findings), recurrent, unresolved spells including transient ischemic attacks, transient global amnesia, cardiogenic syncope, polypharmacy or use of CNS-affecting medications other than selective serotonin reuptake inhibitors, and/or persistent sleep disturbances such as insomnia, hypersomnia, REM behaviour disorder, or moderate to severe obstructive sleep apnea. Ultimately 36 subjects with no clinical history or concern for cognitive impairment and meeting the above exclusion criteria were selected from the same-age cohort and were excluded from the 110 001 adult patients used to create the 12 176 × 19 × 45 tensor. See Table 1 for patient demographic information.

**Table 1:** Characteristics of the EEG dataset and the patient population

Attribute	Statistics
EEG recordings	Unique records: 12.176
	Unique participants: 11 001
Age	Range: 18–101
	Mean = 49.92
	Age groups:
	18–30: 2473
	30–50: 3555
	50–70: 3987
	>70: 2161
Gender	Female = 5966 (54%)
EEG grade	Normal: 6058
	Dysrhythmia 1: 3020
	Dysrhythmia 2: 1369
	Dysrhythmia 3: 1729
Cognitive impairment subgroups	Cognitively Normal (CN)
	*N* = 36
	Age: range = (50–86) mean = 69
	Gender: Female = 17 (47%)
	AD-Mild Cognitive Impairment (AD-MCI)
	*N* = 30
	Age: range = (52–89) mean = 68
	Gender: Female = 14 (47%)
	Alzheimer’s Disease Dementia (ADem)
	*N* = 39
	Age: range = (52–88) mean = 68
	Gender: Female = 25 (64%)
	Dementia with Lewy Bodies (DLB)
	*N* = 31
	Age: range = (54–84) mean = 70
	Gender: Female = 4 (13%)

### Projecting new data onto the space of discovered factors

To estimate the factor weights of the above cohort of EEGs on each of the discovered set of factors in an unbiased way, we utilized a two-dimensional (2D) approximated projection approach.^33^ First, we created a rank-1 matrix for each factor by taking the outer product of the channel and spectral factors, resulting in a matrix of size 19 × 45 (19 channels and 45 frequencies). Next, we unrolled this matrix (corresponding to a factor) into a vector of size 1 × 855. Then, we stacked these vectors to form a matrix of all unrolled factors and solved a linear inverse problem to find the weights for the new data (of size 1 × 19 × 45) on the unrolled set of factors from the original population tensor. If we use *U* to denote this matrix of unrolled factors from the original population tensor (of size 6 × 855 using 6 factors of interest) and *x* to denote the unrolled new data (of shape 1 × 855), then the projected weights are found as *xU*^−1^.

### Classification framework

We then employed a Naïve Bayes (NB) classification approach to evaluate the potential of classifying the different cognitive etiologies from normal controls (i.e. AD-MCI versus CN, ADem versus CN and DLB versus CN). We chose to utilize the NB classifier for this task because of the relatively small sample size in each of those classifications. We identified exactly one EEG per patient (the first EEG in chronological order if multiple EEGs were available for a single patient). We utilized the weights of each factor obtained via tensor decomposition corresponding to the identified EEGs as features in the classification approach. We assigned labels of ‘1’ and ‘0’ to disease and control groups, respectively, and evaluated them using a leave-one-patient-out cross-validation strategy. We utilized the area under receiver operating characteristics curve as the primary measure of classification potential for comparisons.

### Clinical correlations

Next, focusing on the automatically extracted factors that best differentiated cognitively impaired from cognitively normal controls in the NB classifier, we compared Factors 2, 3, 4 and 6 to established clinical markers of disease severity and cognitive performance. Three pair-wise comparisons were carried out for each factor (CN versus AD-MCI, AD-MCI versus ADem and ADem versus DLB), utilizing a Mann-Whitney-Wilcoxon two-sided test with Bonferroni correction (α = 0.05/3). Analysis of Factors 1 and 5 are included in the supplement. As was done in our classification analysis, we accounted for the role of age in EEG findings by regressing the factors against the subject’s age at time of EEG recording. Univariate linear regressions were used in the following comparisons as our goal was simply to determine if the automatically extracted EEG features were biologically meaningful (i.e. showed some correlation with established clinical tests); quantifying the degree to which the extracted features explained variations in clinical exam scores or disease biomarkers through additional statistical modelling was beyond the scope of this proof-of-concept exercise.

The following main analyses were performed at the cohort level, combining ADem, AD-MCI and DLB subjects. As exploratory analyses to evaluate the effect of clinical diagnoses on the association between EEG features and established biomarkers, we repeated the tests within the ADem/AD-MCI and DLB groups separately.

### Kokmen short test of mental Status

We assessed correlations between the automatically extracted EEG features and cognitive exam scores based on the Kokmen STMS. Ninety-seven subjects had recorded Kokmen STMS scores within 12 months of their EEG. First, we performed univariate linear regressions between age-adjusted EEG factors and Kokmen STMS scores for all 92 subjects, *P*-values were adjusted by Bonferroni correction (α = 0.05/4). As exploratory analysis, we performed the same regressions within the clinical DLB group (*n* = 27) and in a subset of the clinical ADem and AD-MCI group that had CSF biomarker measures supportive of AD pathology (abnormal AB42 and/or p-Tau/AB42 ratio) (*n* = 20).

### FDG-PET regional SUVR

Next, we assessed correlations between the automatically extracted EEG features and FDG-PET standardized uptake value ratios (SUVR) at the voxel level. Sixty-two subjects had clinical ^18^F-FDG-PET scans within 12 months of their EEG, which were acquired using a PET/CT scanner (GE Healthcare). Participants were injected with ^18^F-FDG in a dimly lit room and, after a 30-minute uptake period, four 2-min dynamic frames were acquired. PET images were normalized to MNI space and masked using a grey matter template. We then intensity normalized the images to the pons and smoothed with a 5 mm full-width half-maximum Gaussian kernel. Finally, SUVR were standardized to a sample of age- and sex-matched cognitively normal controls (*n* = 492).

Voxel-wise regression analyses of the age-adjusted factor residuals and SUVR were performed for the total sample and, as exploratory analysis, within each subgroup (clinical DLB, *n* = 18 and CSF biomarker supported ADem/AD-MCI, *n* = 16). We additionally assessed the extent to which the association between FDG-SUVR and EEG factor loadings differed between the subgroups by exploring interaction terms of each factor × subgroup as a predictor for the regression analyses. For these analyses, the main terms of the interaction were entered as covariates. Effects are shown unthresholded and thresholded with a false discovery rate corrected alpha level of 0.05. Analyses of FDG-PET and EEG features were carried out using the Nilearn library (version 0.9.0) in python.^41^

### CSF Alzheimer’s disease biomarkers

Finally, we assessed correlations between extracted factors and CSF AD Biomarker measures. Forty-six subjects underwent Roche Elecsys AD CSF Biomarker testing within 12 months of their EEG. We adjusted for the effect of age on CSF biomarker values using the same linear regression method against the subject’s age at the time of CSF testing. Univariate linear regressions were performed between the age-adjusted EEG factor residuals and age-adjusted AD biomarker residuals (AB42, total Tau (tTau) and pTau and the pTau/AB42 ratio). *P*-values were adjusted for repeat comparisons via a Bonferroni correction (α = 0.05/12).

As an exploratory analysis, we repeated the regressions in the clinical ADem/AD-MCI group regardless of CSF biomarker results (*n* = 33) and the DLB group (*n* = 13). We did not further segregate the ADem/AD-MCI subjects based on biomarker normal/abnormal status as biomarker values were the variable of interest.

Statistical analyses for the STMS and AD Biomarker sections were carried out using R version 4.1.1.

## Results

### Data characteristics

Table 1 shows the characteristics of the EEG dataset and the patient population. The EEG dataset included 12 176 unique routine EEG studies from 11 001 unique participants. Most repeat studies were performed on seizure patients evaluating for epileptic foci and antiepileptic drug efficacy, some with upwards of six studies for a single participant. The participants were from a wide age range between 18 and 101 with a mean age of ∼50, with a majority between the ages 30 and 70. In addition, most of the EEG studies (49.8%) were normal based on expert visual review. We further identified an age-matched subset of patients who met the criteria for neurodegenerative etiologies and cognitively normal controls. Specifically, we identified EEG studies of 36 CN, 30 Ad-MCI, 39 ADem and 31 DLB patients for further analyses. All individuals in this subset were within the age range of 50–89. One control subject had two EEGs performed 2 years apart; we used the first EEG which was performed within 12 months of clinical documentation of normal cognition.

### Tensor decomposition produces meaningful factors

**Figure 2:**
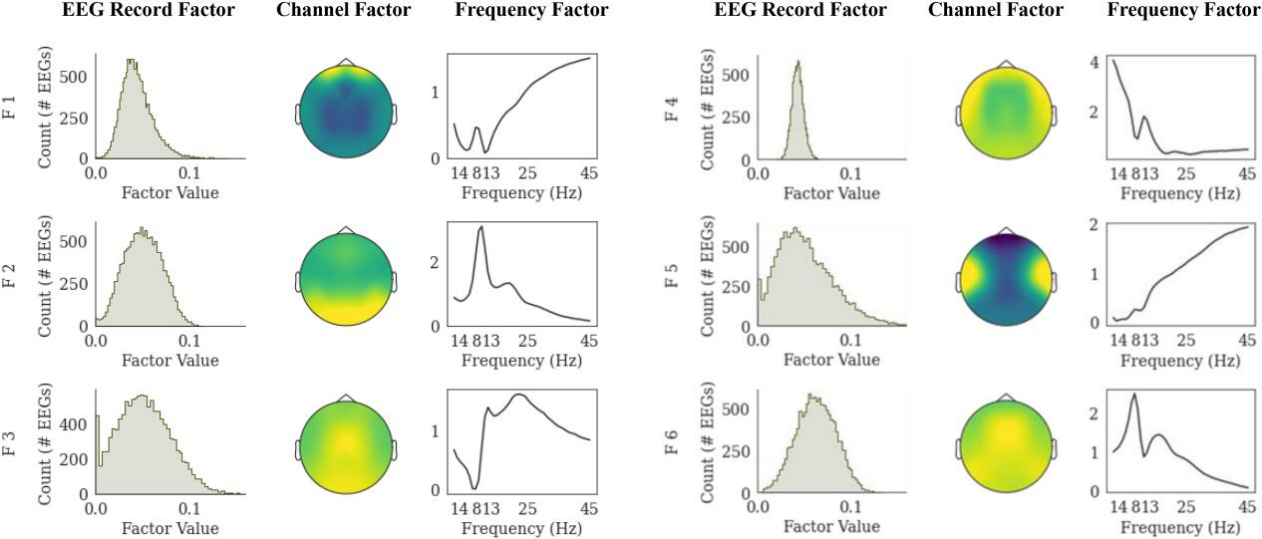
**Factors derived using a nonnegative canonical polyadic decomposition.** A large population-level routine EEG database was used to identify common spectral patterns during eyes-closed wakefulness. Using a nonnegative canonical polyadic decomposition, we decomposed the 19-channel power spectra of eyes-closed epochs extracted from 12 176 routine EEG records into six factors. Each factor consists of three components representing the EEG record, channel and frequency dimensions related to a common brain activity pattern. While the EEG record factor represents the weights of EEGs with respect to the spatial and spectral profiles, we illustrate it using a histogram for clarity. We postulate that factors 1–6 represent eye movement, posterior alpha rhythm, Rolandic beta activity, frontal slowing, bitemporal muscle activity and slower alpha activity, respectively.

Figure 2 illustrates six factors derived using a nonnegative canonical polyadic decomposition of the average power spectral densities extracted from 12.176 EEG during eyes-closed wakefulness. Each factor consists of three rank-1 tensors corresponding to the EEG record, channel and frequency dimensions. Factor 1 indicates beta and gamma (13–45 Hz) activity focused on the prefrontal regions, which might represent eye-movement-related muscle activity. Factor 2 indicates alpha activity (8–13 Hz) in the posterior region, which might represent the posterior dominant alpha rhythm. Factor 3 indicates beta activity (13–25 Hz) in the centroparietal regions, which may be associated with a host of different biological generators,^42^ including Rolandic beta activity from the sensorimotor area.^43^ Factor 4 indicates delta and theta (1–6 Hz) activity in the frontotemporal regions, which could represent frontal slowing associated with cognitive decline.^44^ Factor 5 indicates beta and gamma (13–45 Hz) activity focused on the bitemporal regions, which could represent temporal muscle artefacts. Finally, factor 6 indicates theta to slower alpha band (6–9 Hz) activity focused on the posterior regions, which could represent slowing of the posterior dominant rhythm.

### Factor correlations with age and EEG grade

**Figure 3:**
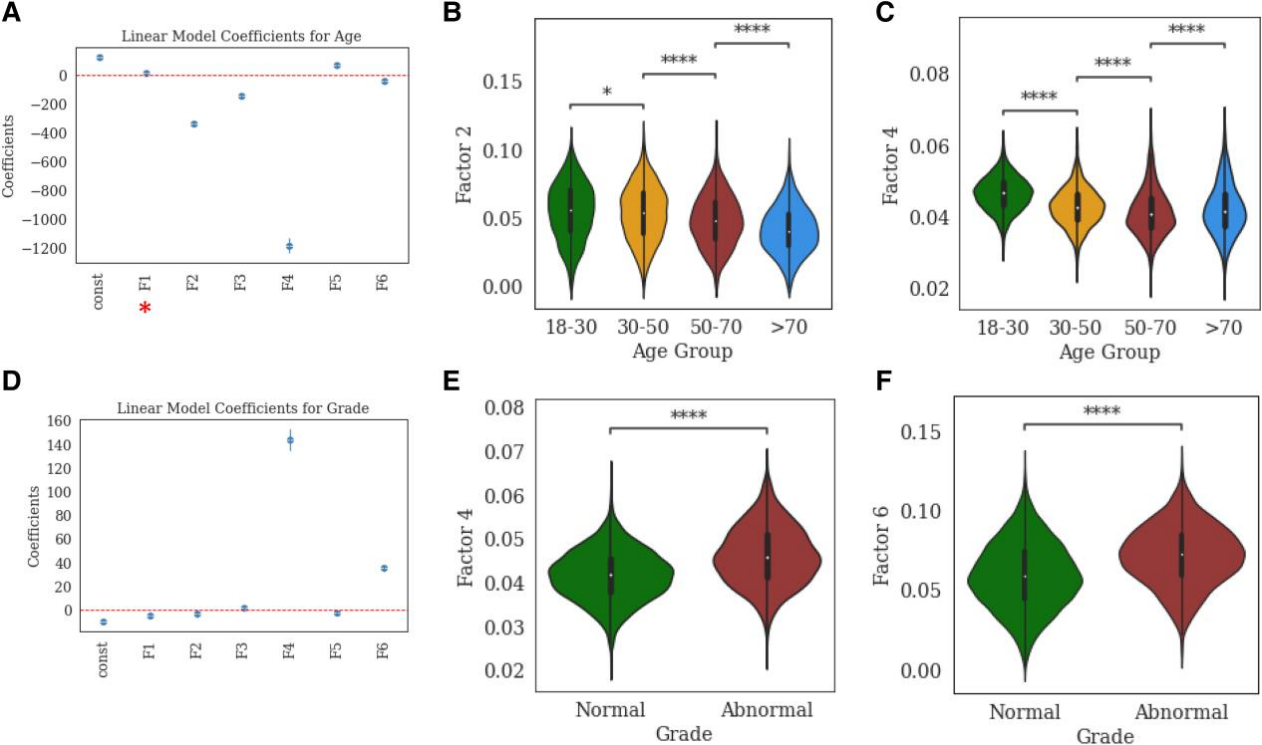
**Factor correlations with age and EEG grade.** (**A**): Linear model coefficients of the factor loadings in explaining patient ages. (**B** and **C**): violin plots showing the distributions of factor 2 and 4 loadings in different age groups. (**D**): Logistic model coefficients of the factor loadings in explaining binarized EEG abnormality grades (normal versus abnormal). (**E** and **F**): violin plots showing the distributions of factor 4 and 6 loadings in normal and abnormal EEG records. In figures A and D, the redline indicates a zero coefficient and the red asterisk indicates non-significant results (*P* > 0.05). In figures B, C, E and F, a non-parametric Mann-Whitney-Wilcoxon test with Bonferroni correction was used for pair-wise comparisons. * indicates a significant difference with *P* < 0.05 and **** indicates a significant difference with *P* < 1e-4. Full cohort of unique EEG recordings (*n* = 12 176). Age group 18–30 (*n* = 2629), 30–50 (*n* = 3742), 50–70 (*n* = 4309), > 70 (*n* = 2370). EEG grade: Normal (*n* = 6476), Abnormal (Dysrhythmia Grade 1–3) (*n* = 6754).

Figure 3 illustrates the relationship of the identified factors with participant age and EEG grade. Here we analyzed the factor loadings with respect to the six factors defined by the channel and frequency dimensions. We performed an ordinary least squares analysis and a logistic regression analysis, respectively, to quantify the relationships of the factors with age and binarized EEG grade (i.e. normal versus abnormal). Figure 3A shows the linear model coefficients for age and boxplots of highly weighted factors in different age groups. Utilizing a Mann-Whitney-Wilcoxon two-sided test with Bonferroni correction (α = 0.05/3) to compare age groups (18–30 versus 30–50, 30–50 versus 50–70, 50–70 versus >70), we found that Factors 2 and 4 were weighted higher compared to other factors in explaining age and that both factors were negatively correlated with increasing age (Fig. 3B and C). Figure 3B shows the linear model coefficients for binarized EEG grade and boxplots of highly weighted factors for different EEG grade categories. We find that Factor 4 was weighted the highest in explaining EEG abnormality grade while Factor 2 showed a moderate correlation (Fig. 3E and F).

### Factors exhibit potential in classifying neurodegenerative etiologies

**Figure 4:**
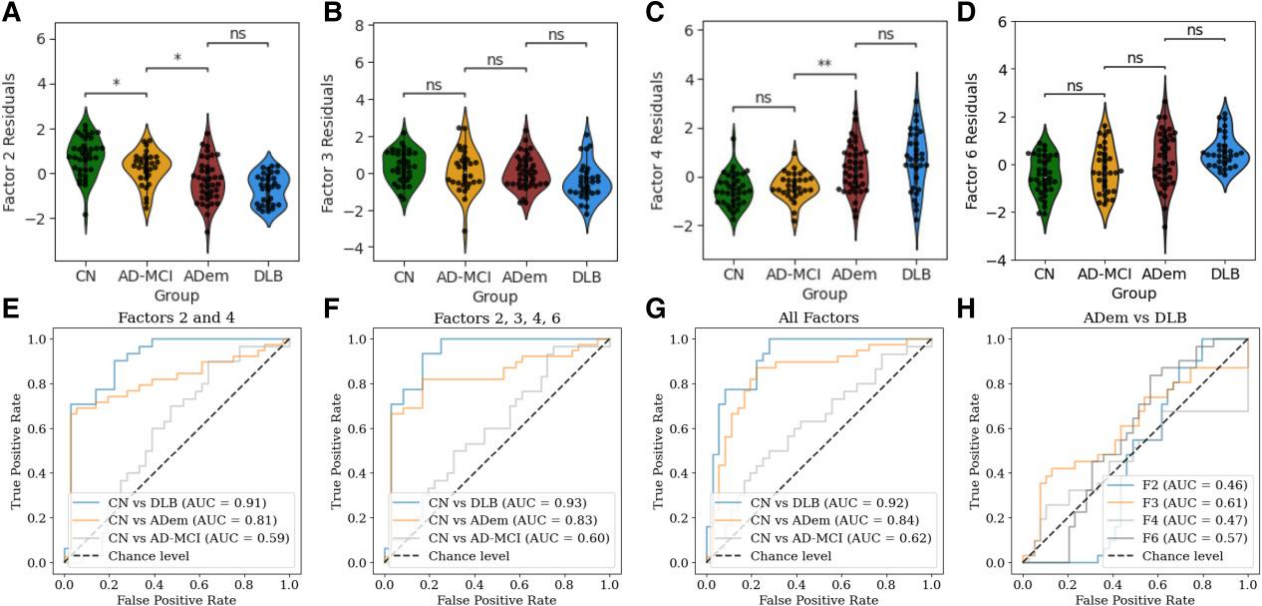
**Differentiation between neurodegenerative diseases and machine learning classification.** (**A–D**): Violin-swarm plots illustrating the differences between cognitively normal (CN) (*n* = 36), Alzheimer’s Disease associated mild cognitive impairment (AD-MCI) (*n* = 30), Alzheimer’s disease dementia (ADem) (*n* = 39), and dementia with Lewy bodies (DLB) (*n* = 31) based on loadings of factors 2, 3, 4 and 6 respectively. (**E–G**): classification of AD-MCI, ADem and DLB patients against CN individuals using leave-one-patient-out cross-validation strategy, using (**E**) factors 2 and 4 only, (**F**) factors 2, 3, 4, and 6, and (**G**) all six factors. (**H**) classification of ADem patients against DLB patients using factors 2, 3, 4 and 6 separately. In figures A-D, a non-parametric Mann-Whitney-Wilcoxon test with Bonferroni correction (α = 0.05/3) was used for pair-wise comparisons. ns indicates no significant difference, * indicates a significant difference with *P* < 0.05 and ** indicates a significant difference with *P* < 0.01.

We then analyzed the relationship of the above factors with neurodegenerative etiologies after controlling for age, the results of which are shown in Fig. 4A–D. Factor 2 (Fig. 4A) and Factor 4 (Fig. 4C) loadings show decreasing and increasing relationships, respectively, with disease severity (cognitively normal controls, MCI, dementia). Factors 2 was able to differentiate the AD-MCI group from cognitively normal controls, and both Factors 2 and 4 were able to differentiate AD-MCI from ADem. Factor 3 (Fig. 4B) demonstrated a similar trend to Factor 2 with lower factor loadings in dementia groups (DLB and ADem) compared to AD-MCI and cognitively normal controls, whereas Factor 6 (Fig. 4D) demonstrated a similar trend to Factor 4, with higher factor loadings in dementia and MCI groups.

We also performed experiments to quantify the classification potential as illustrated in 4D-F. We found that factors 2 and 4 were able to classify ADem and DLB from CN with good AUC metrics (Fig. 4E) and that the addition of factors 3 and 6 (Fig. 4F), or using all the factors did not provide any significant gains in the classification potential (Fig. 4G). Factors 2 and 4 demonstrated moderate AUC metrics in classifying AD-MCI from CN; again, the addition of factors 3 and 6, or all factors did not significantly improve classifying potential. Finally, we found that factors 3 and 6 could classify DLB patients and ADem patients while factors 2 and 4 did not perform better than chance level (Fig. 4H). (For AUC confidence intervals and *P*-values see Supplementary Table 1).

## Clinical correlations

### Kokmen short test of mental Status

**Figure 5:**
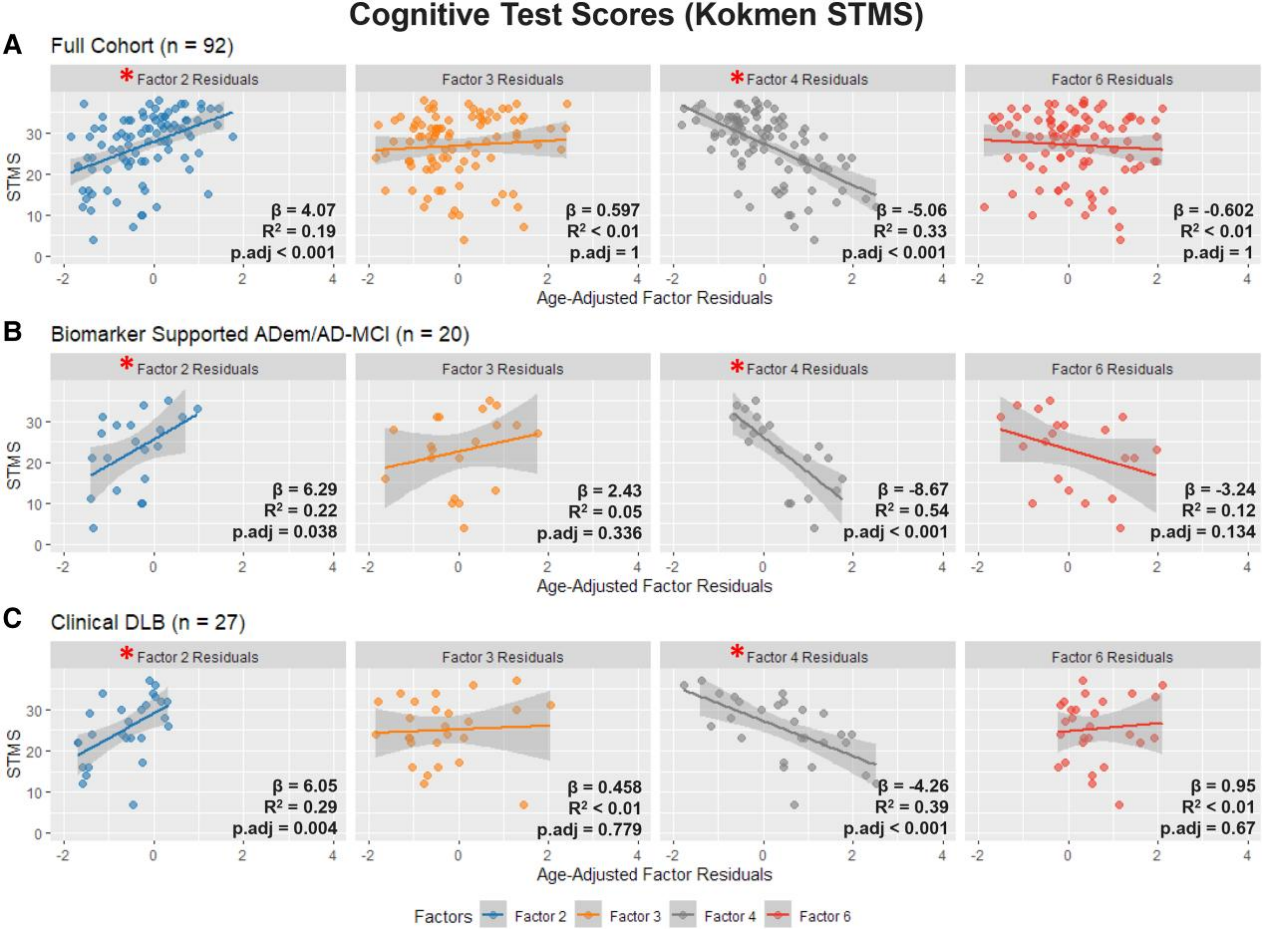
**Univariate linear regression between Kokmen STMS and Factors 2, 3 and 4.** (**A**) For the full cohort (*n* = 92), Factor 2 showed a positive correlation whereas Factor 4 showed a negative correlation with cognitive performance. *P*-values by Bonferroni correction. (α = 0.05/4). (**B**) Exploratory subgroup analysis of biomarker-supported Alzheimer’s Dementia and AD-MCI subjects (*n* = 20), Factor 2 also shows a positive correlation whereas Factor 4 a negative correlation. (**C**) Subgroup analysis of clinical DLB subjects (*n* = 27), Factor 2 again shows a positive correlation whereas Factor 4 shows a negative correlation. * = Statistically Significant Correlation.

Across the full cohort of subjects with a documented STMS within 12 months of their EEG (*n* = 92), Factor 4 demonstrated a negative correlation with STMS scores (β = −5.06, *R*^2^ = 0.33, *p.adj* < 0.001) and Factor 2 demonstrated a positive correlation with STMS scores (β = 4.07, *R*^2^ = 0.19, *p.adj* < 0.001). Factors 3 and 6 showed no correlations with cognitive exam scores. (Figure 5A) No significant correlations were found between Kokmen STMS scores and Factors 1 and 5 at the cohort level. (Supplementary Fig. 1).

**Figure 6:**
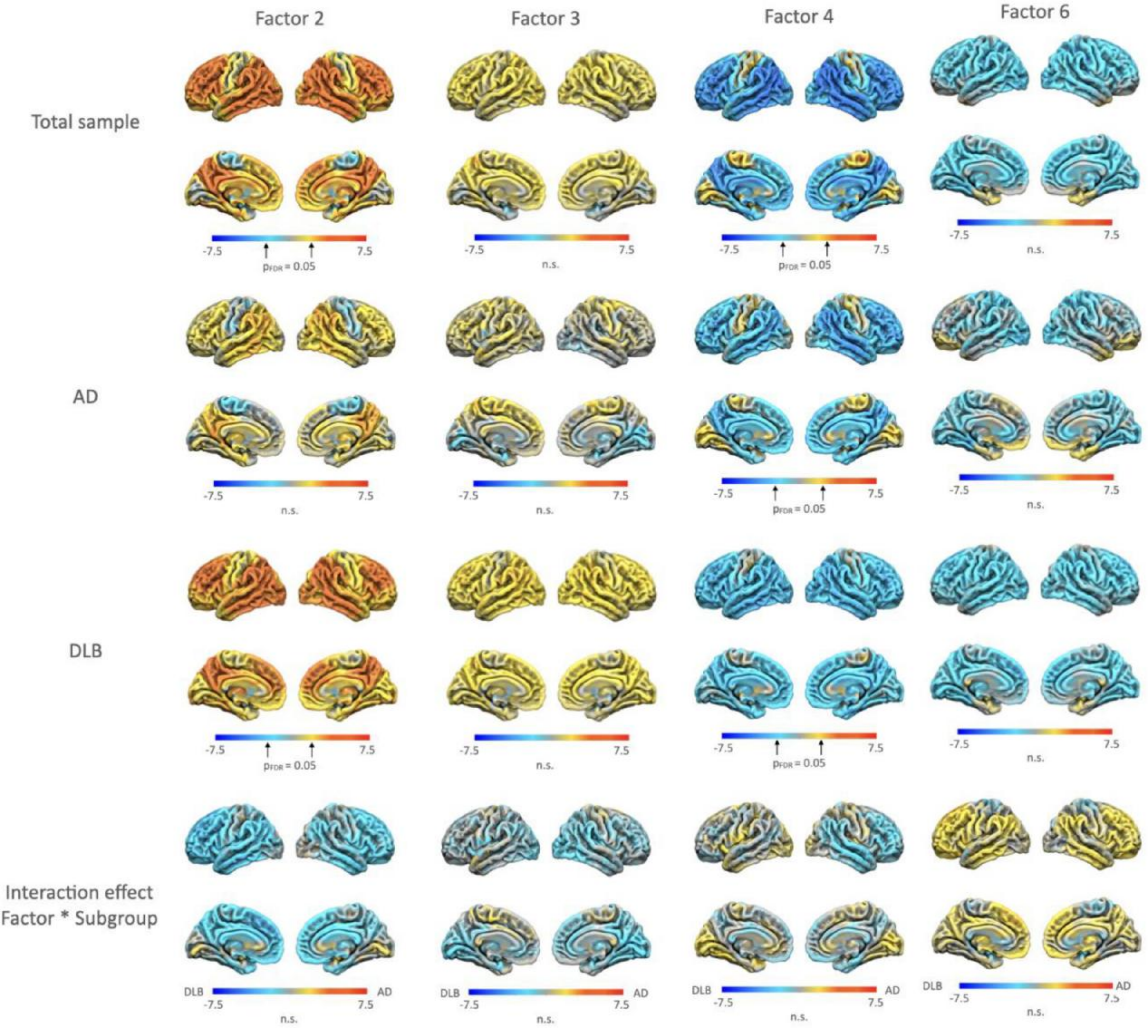
**Z-maps of voxelwise regression analyses between FDG-PET SUVR and Factors 2, 3 and 4.** For the whole sample, AD and DLB subgroups, and interaction effects of the factors × subgroups. For the interaction effects, warmer colours (red) indicate greater effects for the AD, colder colours (blue) greater effects for the DLB subgroup. Z-maps thresholded with a false discovery rate corrected *P* = 0.05 can be found in the Supplementary material (Supplementary Fig. 2). Total sample (*n* = 62), CSF biomarker supported ADem/AD-MCI (*n* = 16), clinical DLB (*n* = 18).

Exploratory analysis demonstrated that the correlation between Factors 2, 4 and STMS score remained robust in both the DLB subgroup and the biomarker-supported ADem/AD-MCI subgroup. Factors 3 and 6 failed to show any correlations with STMS scores in either subgroup. (Figure 6B and C)

### FDG-PET regional SUVR

Voxel-wise regression analyses with FDG-PET SUVR showed positive correlations in lateral frontal, parietal temporal, occipital regions for Factor 2 across the whole sample with available FDG-PET, whereas Factor 4 demonstrated negative correlations in the same regions (*n* = 62). For Factor 3 we observed similar, albeit weaker, associations to Factor 2 and Factor 6 demonstrated similar, but weaker correlations to Factor 4. (Figure 6. See Supplementary Fig. 2 for effects thresholded with FDR-corrected *P* = 0.05). Regressing voxel-wise effects for Factor 4 on Factor 2 effects showed that effects were an inverse of each other (Supplementary Fig. 3).

Repeating the analyses in each subgroup and using interaction analyses demonstrated that effects for Factor 2 were mostly driven by the DLB subgroup. Factor 4 effects were not significantly different between the ADem/AD-MCI and DLB subgroups. For Factors 1 and 5 we only observed weak effects (Supplementary Fig. 4).

### CSF Alzheimer’s disease biomarkers

**Figure 7:**
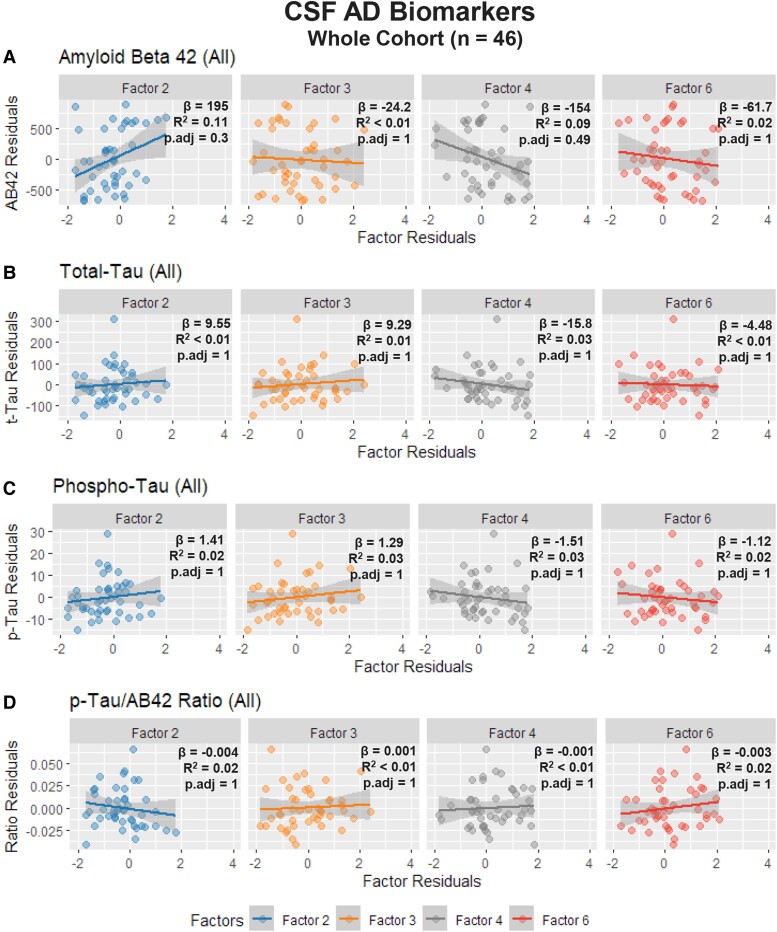
**Univariate linear regression between age-adjusted Factors 2, 3, 4, 6 and Alzheimer’s Disease Cerebrospinal Fluid Biomarkers.** (**A–D**) Full Cohort (*n* = 46). For the full cohort, Factor 2 demonstrated a positive correlation and 4 demonstrated a negative correlation with Amyloid Beta 42 measures but failed to survive correction for multiple comparisons (**A**).

Across the full cohort of subjects with AD CSF biomarker results (*n* = 46), univariate linear regression analysis demonstrated a positive trend between Factor 2 and CSF AB42 measures (*β* = 195, *R*^2^ = 0.11, *p.adj* = 0.3) and a negative trend between Factor 4 and CSF AB42 measures (*β* = −154, *R*^2^ = 0.09, *p.adj* = 0.49). Both correlations failed to survive correction for multiple comparisons. (Figure 7A) No significant correlations were found between CSF biomarker measures and Factors 1, 3, 5 and 6 at the cohort level. (Figure 7A and Supplementary Fig. 6).

**Figure 8:**
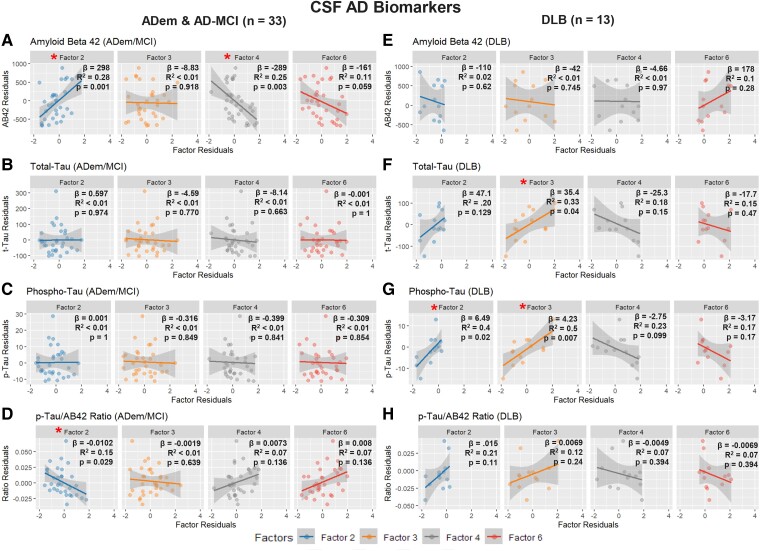
**Univariate linear regression between age-adjusted Factors 2, 3, 4, 6 and Alzheimer’s Disease Cerebrospinal Fluid Biomarkers.** (**A–D**) Clinical Alzheimer’s Dementia and Mild Cognitive Impairment (ADem & AD-MCI). (**E–H**) Dementia with Lewy Bodies (DLB). In the ADem & AD-MCI subgroup, Amyloid Beta 42 demonstrated a positive correlation with Factor 2 and a negative correlation with Factor 4 (**A**), driving the association between the p-Tau/AB42 ratio and Factor 2 (**D**). In the DLB subgroup, Total-Tau (**F**), phosphorylated-tau (**G**) and the p-Tau/AB42 Ratio (**H**) demonstrated a positive trend with Factors 2 & 3, and a negative trend with Factors 4 & 6. Only the Factor 3 association with Total-Tau (**F**) and Factors 2 & 3 associations with phosphorylated-Tau (**G**) reached statistical significance. (* = Statistically Significant Correlation).

Exploratory analyses of the ADem/AD-MCI subgroup revealed a more robust negative correlation between Factor 4 to CSF AB42 correlation (*β* = −289, *R*^2^ = 0.25, *P* = 0.003) and a positive correlation between AB42 and Factor 2 (*β* = 298, *R*^2^ = 0.28, *P* = 0.001). We found no significant associations between the factors and p-tau levels in this subgroup, suggesting that associations between the p-Tau/AB42 ratio and Factor 2 was driven by the AB42 associations (Fig. 8A and D).

In the DLB subgroup, p-Tau residuals showed a robust positive correlation with Factor 2 (β = 6.49, *R*^2^ = 0.4, *P* = 0.02) and Factor 3 (β = 4.23, *R*^2^ = 0.5, *P* = 0.007). Factor 4 and 6 showed an opposite, negative trend with p-Tau but failed to reach significance. Total-Tau showed a similar positive trend with Factors 2 and 3 (with only Factor 3 reaching statistical significance), and a negative trend with Factor 4, as did the p-Tau/AB42 ratio, which was driven by the association with p-Tau. No correlations were noted with AB42 measures (Fig. 7I–L).

## Discussion

In this study, we performed a large-scale tensor decomposition of population-level routine EEG data set containing 12 176 EEG records and identified meaningful features representing known brain activity patterns in EEG recordings during eyes-closed wakefulness. The automatically extracted features demonstrated high classification accuracies between cognitively normal and MCI and especially dementia subjects and to a lesser extent between ADem and DLB. Features which approximated posterior alpha, anterior theta-delta and centroparietal beta activity correlated with established markers of disease severity including STMS, FDG-PET SUVR and CSF AB42 concentration. These studies demonstrate that coupling routine clinical EEGs with automated, data-driven analysis methods could lead to a quick, noninvasive and relatively inexpensive test to differentiate between different neurodegenerative causes of cognitive impairment.

### Modular and explainable features derived using tensor decomposition

Several previous studies have investigated the use of tensor decomposition on multichannel EEG data.^22^ Unlike the traditional approaches that focus on predetermined frequency bands such as the delta, theta, alpha, beta and gamma bands, the tensor decomposition approach can derive frequency and spatial profiles in a data-driven manner. Some groups have utilized tensor decomposition on ictal EEG data to accurately localize seizure onset zones in focal epilepsy patients.^45,46^ Others have utilized tensor decomposition on event-related potentials (ERP) to characterize disease states^47^ and differences between stimuli.^48^ In the latter ERP studies, tensors were generally formed using time, frequency, space and group dimensions. In the area of neurodegenerative diseases, EEG tensor decomposition has been explored previously, including comparisons between CN, MCI and ADem.^23,49^

In relation to these AD EEG studies, our work presents the first analysis using a large-scale population-level EEG database characterizing the main brain activity patterns during eyes-closed wakefulness. Notably, the modular decomposition successfully extracted well-known biologically meaningful brain activities (e.g. posterior alpha rhythm, anterior slowing, centroparietal beta) without having to first reject segments associated with eye blink or temporalis muscle artefact contamination. As such, our study presents an unbiased approach to the identification of EEG spectral and spatial profiles and has the potential to find additional patterns by analyzing EEG segments associated with other physiological and pathological states.

### Early identification of cognitive impairment

Our results indicate that our tensor decomposition approach of resting state EEG has substantial classification potential to distinguish cognitively normal subjects and those with cognitive impairment due to underlying Alzheimer’s or Lewy Body pathology. The classification potential of using Factors 2 and 4 also appears to extend to patients with mild cognitive impairment due to Alzheimer’s disease (AD-MCI) (AUC 0.59), defined here as having a Kokmen cognitive test score greater than 30 (out of 38) and meeting the NIA-AA diagnostic guidelines for AD.^25^

Early identification of cognitive impairment, particularly in the preclinical or MCI stages is crucial for clinical prognostication and risk stratification, which in turn inform counselling and selection of potential treatments. While several previous studies already demonstrated this potential using resting state EEG,^10,11^ the clinical applicability of these methods is ultimately dependent on incorporating these techniques into a routine clinical workflow. The tensor decomposition methods explored here can be used to automate the extraction of EEG features that correlate with biologically meaningful electrophysiologic attributes, and may ultimately lead to a more economical option for early identification of individuals with MCI or dementia compared to current methods of CSF biomarker testing, FDG-PET and neuropsychologic exams.^50^

### Associations with established clinical markers

Our results demonstrate strong associations between data-driven EEG features and dementia physiology, and further support the purported biological meaning behind the automatically extracted factors. Higher Factor 4 and lower Factor 2 loadings were associated with worse pathologic disease states (i.e. lower Kokmen STMS and FDG-PET metabolism), consistent with established literature demonstrating that an increase in slow wave (delta-theta) activity^44^ and a reduction in posterior alpha activity,^51^ respectively, are associated with increasing dementia severity and cognitive decline. Factor 6, which corresponds to slower theta-alpha band activity in the same posterior region as Factor 4, demonstrates similar higher loading with lower cognitive test performance and FDG-PET hypometabolism.

Factor 3 is also positively associated with FDG-PET metabolism, which appears to be driven by the DLB subgroup but fails to reach statistical significance. Previous work in Parkinson’s disease (PD) patients have shown that motor cortex beta oscillations can be normalized with dopaminergic medication or basal ganglia deep brain stimulation.^52^ Given the shared alpha-synuclein pathology and basal ganglia/dopamine deficits between PD and DLB, this may explain why the Rolandic beta activity represented by Factor 3 is associated with disease severity in DLB but not ADem/AD-MCI and why Factor 3 may uniquely differentiate AD from DLB on machine learning classifiers (AUC = 0.61). Notably, the brain regions associated with EEG factor loadings on FDG-PET are similar to a pattern of lateral frontal-parietal-temporal metabolism representative of global brain functioning in multiple neurodegenerative conditions including ADem, DLB and frontotemporal dementias based on prior work by our group in FDG-PET eigenbrain decompositions.^4^ This suggests that extracted EEG features may offer a non-regional approach to measuring brain function and can complement existing localization-based approaches such as MRI and FDG-PET.

Subgroup analysis also revealed that AD subjects had strong associations between Factors 2 and 4 and CSF AB42 measures whereas the DLB subjects did not, suggesting that beta-amyloid burden is associated with electrophysiologic pathology in ‘pure’ AD cases,^1^ whereas in clinical DLB subjects, the electrophysiologic profile may be driven by α-synuclein regardless of amyloid pathology burden.^9^ The DLB population also demonstrated stronger associations with Factor 2 loadings on FDG-PET, consistent with multiple studies demonstrating disruptions in posterior alpha activity were more associated with Lewy body compared to AD pathology.^9,53^ Combined with DLB’s (but not AD’s) strong association with Factor 3, this suggests that automatically extracted EEG features may be differentially affected by underlying disease pathology and supports its potential role in distinguishing between AD and DLB.

Finally, to our knowledge, no study has evaluated the association between CSF-tau and EEG findings in DLB. In our DLB group, higher Factors 2 and 3 and lower Factors 4 and 6, associated with lower disease severity on all other clinical measures, were associated with increasing t-Tau, p-Tau and p-Tau/AB42 ratio. One possible explanation is that high CSF p-Tau and t-Tau in the DLB subgroup do not necessarily represent a more advanced disease state, but rather a higher contribution of AD pathology to the overall disease burden. DLB subjects with low CSF tau who derive their disease primarily from α-synuclein therefore have worse electrophysiologic disturbances compared to patients whose disease may be attributed to higher co-morbid AD pathology (high CSF Tau). This would be internally consistent with the lack of association between Factor loadings and CSF p-Tau and t-Tau in the ADem/AD-MCI group, and the stronger association between clinical DLB and electrophysiologic disturbances.^7^

### Limitations/future directions

Limitations to the current study methodology are best categorized between EEG processing and subject and biomarker selection.

Since tensor decomposition is a type of blind source separation approach, it may be useful to think of the factors derived from EEG data as distinct physiological origins of EEG activity. Estimates from automated approaches to determine the optimal number of factors often do not conform to the physiological properties of EEG data.^22^ Using a manual trial-and-error visual review approach, we were able to identify the six features representing commonly known sources of EEG activity during eyes-closed wakefulness.^54,55^ We note that this approach may not be feasible in settings with limited existing knowledge of underlying EEG characteristics, and automated approaches, such as difference of fit (DIFFIT)^56^ and automatic relevance determination^57^ could ensure reproducibility and wider adoptability in future studies. Future studies directly comparing data-driven approaches against traditional EEG analysis methods will help determine whether automated approaches are truly robust enough to identify meaningful effects without manual artefact rejection.

Furthermore, we demonstrated the classification potential of the identified features using a simple Naïve Bayes classifier. Naïve Bayes classification is well suited for small sample sizes and independent input features. Although the factors identified by CP decomposition are not necessarily orthogonal by their construct, from visual inspection, we can confirm that the factors represent independent physiological features. Hence, we believe Naïve Bayes is a sensible choice for our classification studies with limited sample sizes. In the future, with larger sample sizes, we may explore additional classification approaches that enable learning of more sophisticated relationships between the factors to further inform our understanding of the underlying physiological processes and their relation to cognitive diseases.

Subject grouping based on clinical presentation and STMS scores rather than biomarker positivity can produce discrepancies between the consensus diagnoses and the underlying pathophysiology. However, the clinical classification scheme does capture the natural heterogeneity of disease pathology seen in neurology clinics. For example, in the clinical DLB subgroup, 5 of 13 subjects (40%) had an abnormal p-Tau/AB42 ratio supportive of AD co-pathology, similar to the reported frequency of AD co-pathology in DLB patients,^39^ whereas 7 of 33 subjects (22%) in the clinical AD-MCI/ADem group had all normal CSF biomarkers and ratios, likely representing some early/mild amnestic cases or tau-negative dementias such as hippocampal sclerosis causing amnestic MCI.^58^ Moreover, establishing correlations between EEG features, underlying neurophysiological processes, and widely used clinical metrics (e.g. clinical consensus criteria and brief cognitive tests) goes toward our goal of making EEG interpretation in neurodegenerative cases more accessible to the community neurologist.

Future studies will utilize true control subjects and better characterized clinical cohorts with pathology confirmation and neuropsychiatric measures, explore different sleep stages, spectral and temporal EEG features and tensor decomposition methods.^59-61^ Longitudinal studies will help evaluate the ability of EEGs to monitor cognitive improvement or response following pharmacologic interventions^62,63^ and in identifying individuals at risk of developing cognitive impairment. Finally, feasibility studies will evaluate how best to integrate these approaches into clinical practice and assess for advantages over existing diagnostic methods in real-world clinic settings.

## Conclusion

This study has demonstrated the ability to reliably differentiate between cognitively normal individuals and individuals with AD and LBD-related cognitive impairment using tensor decomposition of routine clinical 10–20 scalp EEG studies. Our method of automated processing and tensor decomposition of routine clinical scalp EEGs can extract recognized biologically meaningful electrophysiologic features associated with established markers of disease severity and can incorporate these features into group-level clinical classification. This study demonstrates that using clinical EEGs in the diagnosis and management of cognitive impairment is feasible and may significantly improve patient access to timely diagnosis and quality of memory care in community clinical settings.

## Supplementary Material

fcae227_Supplementary_Data

## Data Availability

Summary data can be made available by the corresponding author upon reasonable request.
