# Supplementary material for: Data-driven retrieval of population-level EEG features and their role in neurodegenerative diseases
Source: Brain Commun. 2024 Jul 31;6(4):fcae227. doi: 10.1093/braincomms/fcae227 (PMC11289732; doi:10.1093/braincomms/fcae227)
Supplement: fcae227_Supplementary_Data [file fcae227_supplementary_data.pdf]

Supplementary Materials

	Factors 2 + 4	Factors 2, 3, 4, 6	All Factors
**CN vs DLB**			
AUC	0.91	0.94	0.92
CI	0.84-0.98	0.87-0.99	0.85-0.98
p-value	0.04	0.03	0.03
**CN vs ADem**			
AUC	0.81	0.83	0.84
CI	0.71-0.92	0.74-0.93	0.75-0.94
p-value	0.05	0.05	0.05
**CN vs AD-MCI**			
AUC	0.59	0.6	0.62
CI	0.45-0.73	0.46-0.74	0.48-0.75
p-value	0.07	0.07	0.07

	Factor 2	Factor 3	Factor 4	Factor 6
**ADem vs DLB**				
AUC	0.46	0.61	0.47	0.57
CI	0.31-0.6	0.47-0.75	0.32-0.62	0.44-0.71
p-value	0.07	0.07	0.08	0.07

Supplementary Table 1: AUC, Confidence Interval, and p-value of classification analysis using a Naïve Bayes classifier. Top: Between AD-MCI, ADem, and DLB patients against CN individuals using factors 2 and 4 only versus all six factors. Bottom: Classification of ADem patients against DLB patients using factors 2,3, and 4 separately.

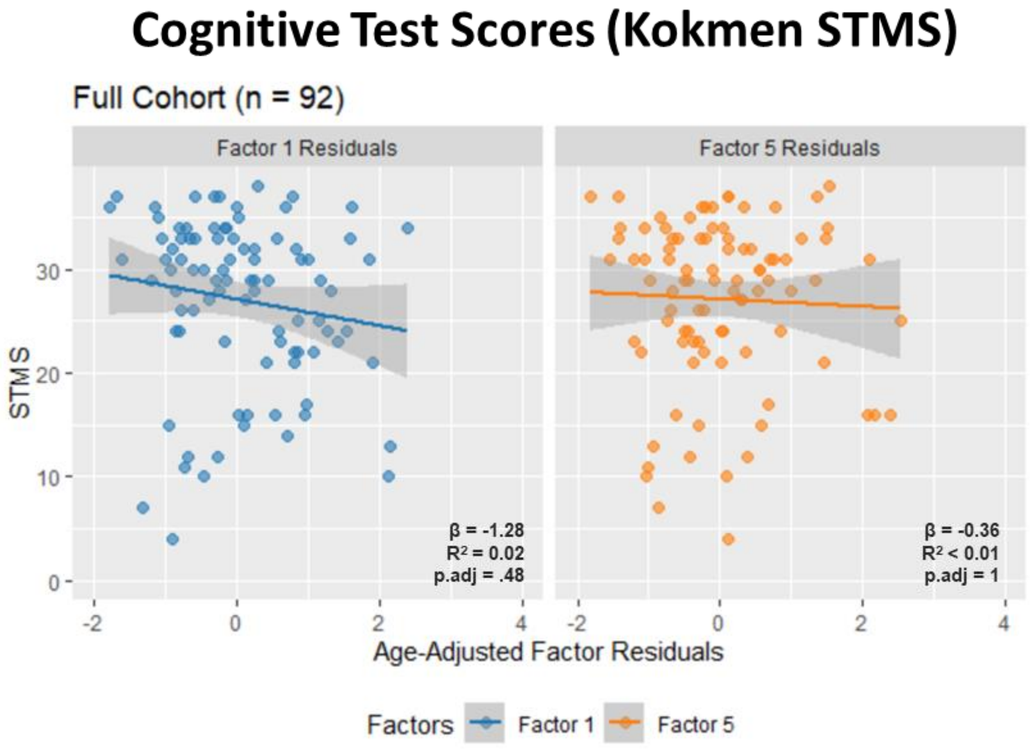

Supplementary Figure 1: No significant correlations were found between Factors 1 and 5 and Kokmen Short Test of Mental Status Scores at the cohort level (n = 92) using univariate linear regression models.

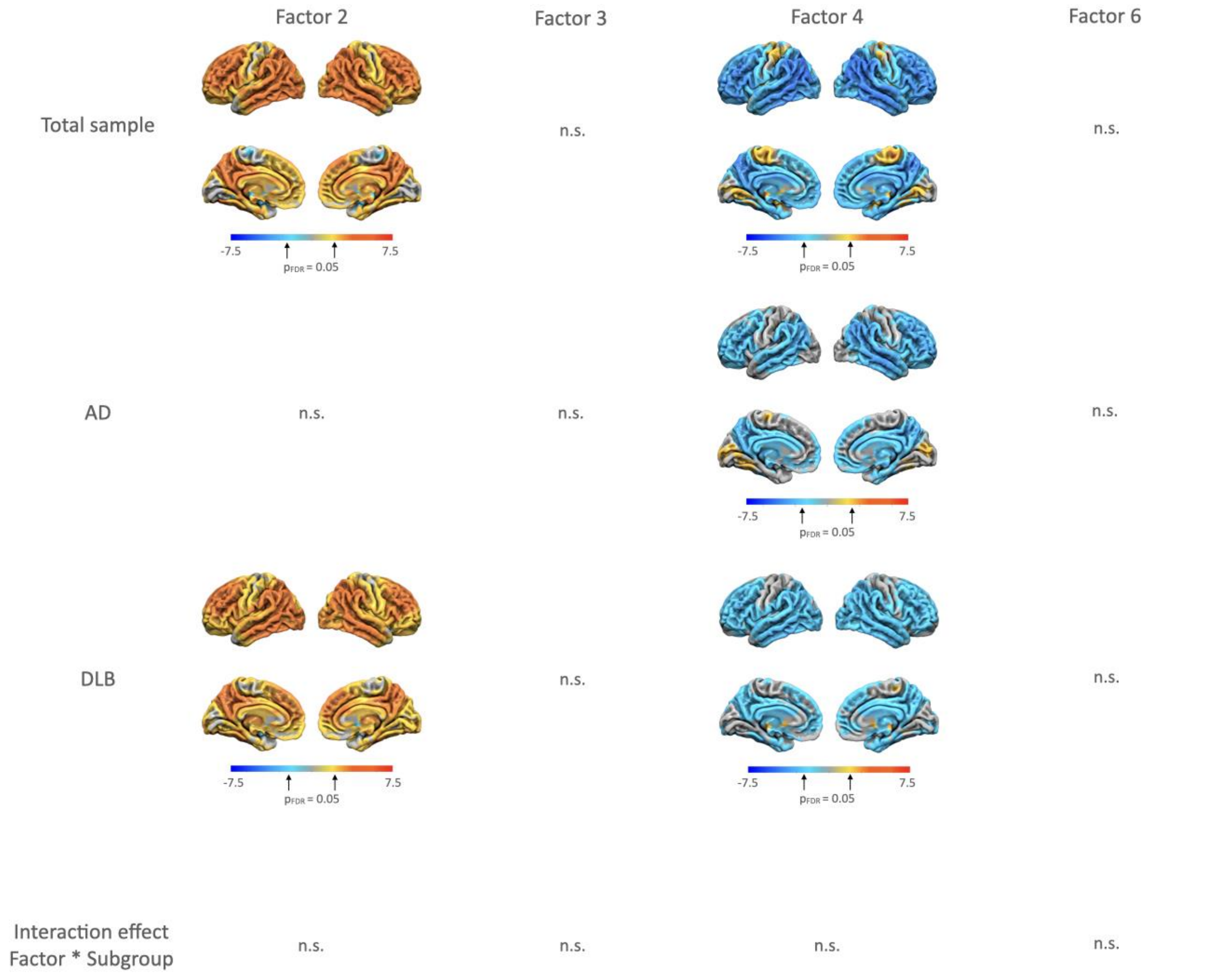

Supplementary Figure 2: Z-maps of voxelwise regression analyses between FDG-PET SUVR and Factors 2, 3, 4, and 6 for the whole sample, AD and DLB subgroups and interaction effects of the factors × subgroups. Effects are false discovery rate corrected with p=0.05. For the interaction effects, warmer colors (red) indicate greater effects for the AD and colder (blue) for the DLB subgroup. Total sample (n = 62), CSF biomarker supported ADem/AD-MCI (n = 16), clinical DLB (n = 18).

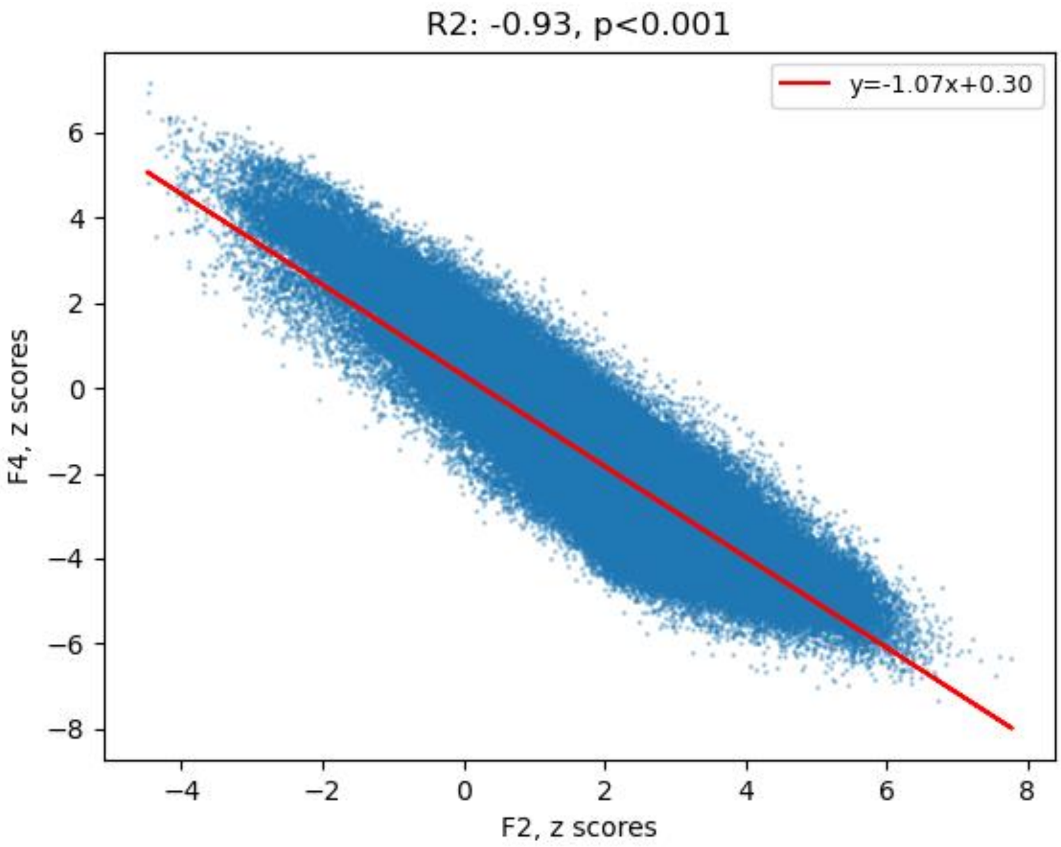

Supplementary Figure 3: Linear regression of voxelwise effects for Factor 4 on Factor 2. Effects were noted to be inverses of each other. Total sample (n = 62)

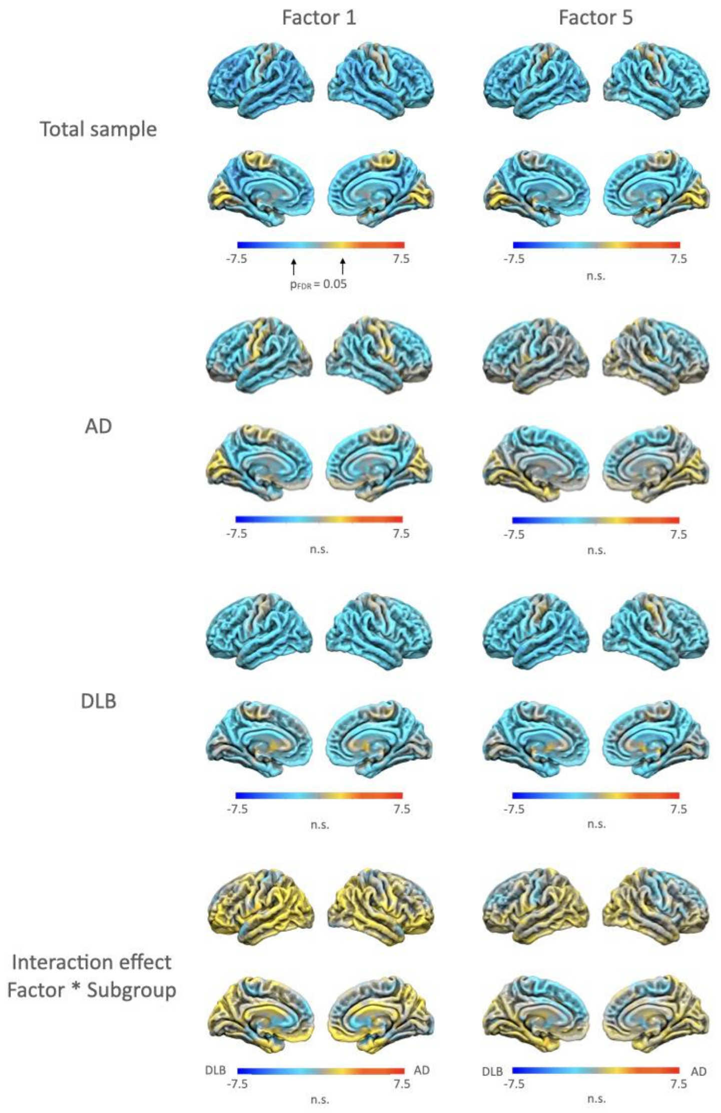

Supplementary Figure 4: Z-maps of voxelwise regression analyses between FDG-PET SUVR and Factors 1 and 5 for the whole sample, AD and DLB subgroups and interaction effects of the factors × subgroups. For the interaction effects, warmer colors (red) indicate greater effects for the AD and colder (blue) for the DLB subgroup. Z-maps thresholded with a false discovery rate corrected p=0.05 can be found in the Supplementary Material (Supplementary Fig. 5). Total sample (n = 62), CSF biomarker supported ADem/AD-MCI (n = 16), clinical DLB (n = 18).

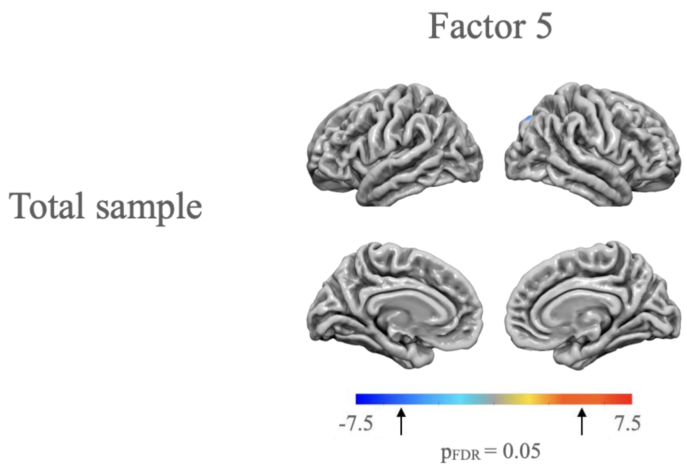

Supplementary Figure 5: Z-maps of voxelwise regression analyses between FDG-PET SUVR and Factor 5 for the whole sample. Effects are false discovery rate corrected with p=0.05. None of the other effects for the subgroups, and for Factors 1 and 6 survived the corrections. Total sample (n = 66)

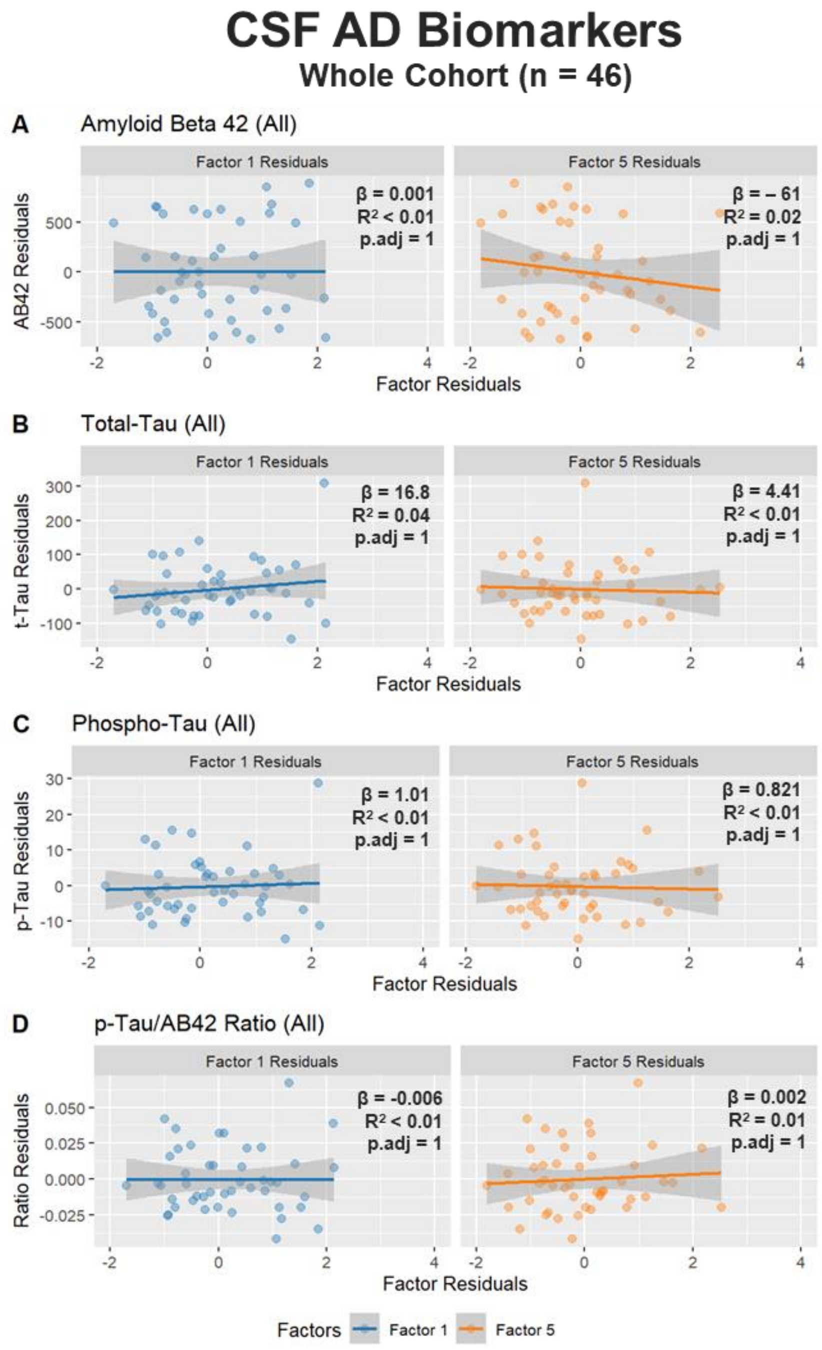

Supplementary Figure 6: No significant correlations were found between Factors 1, 5, and CSF measures of AB42 (A), t-Tau (B), p-Tau (C), or the p-tau/AB42 ratio (D) at the cohort level (n = 46) using univariate linear regression models
